# Evaluating Parenting Stress and Identifying Influential Factors in Caregivers of Deaf and Hard-of-Hearing Children

**DOI:** 10.3390/audiolres15050120

**Published:** 2025-09-20

**Authors:** Yuan Chen, Xiaoli Shen, Chengao Lyu

**Affiliations:** 1 Department of Special Education and Counselling, The Education University of Hong Kong, Hong Kong SAR, China; s1149317@s.eduhk.hk; 2 School of TCM and Pharmacology Health and Early Childhood Care, Ningbo College of Health Sciences, Ningbo 315100, China; shenxiaoli@nchs.edu.cn

**Keywords:** parenting stress, self-compassion, social support, hearing aids, cochlear implants

## Abstract

Parenting stress significantly affects caregivers of deaf and hard-of-hearing (DHH) children, influenced by unique challenges and stressors. **Background/Objectives**: This study aims to develop the Chinese Family Stress Scale (CFSS) and to identify the stressors and contributing factors to elevated stress levels. **Methods**: The study involved 257 caregivers of DHH children aged 0–12 years old. The CFSS was used to assess parenting stress in caregivers of DHH children, with its reliability and validity evaluated. Factors such as speech intelligibility, oral language use, self-compassion, and social support were examined for their impact on parenting stress. **Results**: Key stressors included financial issues, discipline, education concerns, medical care, and safety. Elevated parenting stress was significantly associated with poor speech intelligibility of the child, inadequate oral language use, negative aspects of self-compassion, and insufficient social support. The CFSS showed good reliability and validity in measuring parenting stress among caregivers of DHH children. **Conclusions**: The CFSS is an effective tool for assessing parenting stress in caregivers of DHH children. Interventions to reduce parenting stress can focus on improving children’s communication skills, enhancing caregiver self-compassion, and bolstering social support networks.

## 1. Introduction

Stress related to parenting is an unavoidable psychological response that arises from the responsibilities of parenthood. It constitutes one of the most pervasive worries that caregivers routinely confront [1]. Evidence has shown that caregivers who experience elevated levels of parenting stress often resort to harmful parenting techniques such as physical punishment, child neglect, and abuse [2]. This type of stress can also lead to negative repercussions for the caregiver, including depressive symptoms, dissatisfaction with their parental role, and diminished self-esteem [2]. Furthermore, research has substantiated a link between parenting stress and adverse developmental outcomes in children, such as impaired social-emotional functioning and language skills [3].

### 1.1. Parenting Stress in Caregivers of DHH Children

Diagnosis as deaf and hard of hearing (DHH) represents one of the most common congenital conditions identified at birth [4,5]. It poses a significant barrier to communication and development of the parent–child relationship, especially in hearing parents. However, mixed findings were found on whether parenting stress in caregivers of DHH children was significantly elevated compared to caregivers for children with typical hearing. Some studies reported higher levels of parenting stress [6,7] while others found no significant difference [8,9,10].

The variability in these findings may be partly explained by the test used. Context-specific measures, such as the Family Stress Scale (FSS) [6], are more sensitive to the stressors experienced by caregivers of DHH children, compared to the general measures, such as the Parenting Stress Scale [11]. All studies found statistically significant elevations in parenting stress using FSS, a context-specific measure [6,7]. Currently, the FSS is the only context-specific measure for both children with hearing aids and children with cochlear implants. It evaluates both general (i.e., finances, discipline) and context-specific stressors (i.e., communication, device operation) in parents of DHH children. Top stressors reported by English-speaking caregivers include communication, finances, education concerns, safety, and keeping hearing devices on and in working order [12]. In addition, the parents of contemporary cochlear implant (CI) recipients were found to experience significantly less stress compared to those of children who made use of earlier—generation CI technology [13]. However, the Chinese version is not available. The first purpose of this study was to develop and validate a Chinese version of FSS (CFSS). Parents in Mainland China tend to lack medical and audiological services, have more financial issues, receive less support from society, and put more emphasis on oral mode of communication and academic performance compared to their English-speaking peers [14,15].

Based on Hofstede’s Cultural Dimensions Theory, modern nations can be distinguished by the cultural dichotomy of individualism and collectivism [16]. For example, the United States represents an individualistic society, while China embodies a collectivist one. This cultural framework is employed here to contextualize broad cultural patterns and, specifically, to explore how cultural values may shape perceptions of developmental needs among DHH children, building on Warner-Czyz et al.’s [17] focused examination of DHH populations. In individualistic societies, there is a tendency to prioritize personal achievements of DHH children, such as their communication skills and ability to engage independently in the community [17]. In contrast, collectivist societies often emphasize communal aspects of DHH children’s lives, such as their ability to conform to group norms, manage behavior, and maintain relationships with others [17]. Applying this cultural lens to DHH populations, we propose that such differences in cultural values may drive cross-cultural variations in the primary sources of parenting stress. To explore this possibility, the stressors measured by the FSS were compared between Chinese-speaking and English-speaking caregiver populations of DHH children. We hypothesized that the core stressors experienced by caregivers would differ significantly between these two cultural groups.

### 1.2. Factors Affecting Parenting Stress

Another explanation for mixed findings in perceived stress among caregivers of DHH children can be family vulnerability or resilience, referring to the family’s capacity to withstand and receivable stress. It includes the family’s internal strengths and external systems. According to the Family Adjustment and Adaptation Response Model, a framework developed by Hamilton McCubbin and Joan Patterson to understand how families react to stressful situations or events, families are constantly in a process of adaptation and adjustment in response to stressors. The adaptation process involves a balance between the demands placed on the family (stressors and strains) and the family’s capability to meet these demands (resources and coping strategies). When the demands exceed the capabilities, the family may experience a crisis. On the other hand, if the family has sufficient resources and coping strategies to manage the demands, they can adapt successfully.

Several factors have been identified in previous studies that contribute to the communication skills of children and the stress levels in parents of deaf children. These factors include the age at which the child receives a hearing aid or cochlear implant, maternal education level, the duration of device use, poor spoken language outcomes, and demanding behaviors of the child [13,14,15]. However, the effects of family capability on parental stress have not been fully examined. It was hypothesized that family capability factors, such as self-compassion and social support, are significantly related to the parenting stress of DHH children.

Self-compassion is “being open to and moved by one’s own suffering, experiencing feelings of caring and kindness toward oneself, taking an understanding, non-judgmental attitude toward oneself, taking an understanding, non-judgmental attitude toward one’s inadequacies and failures, and recognizing that one’s inadequacies and failures” [18]. Existing literature suggests that self-compassion is an effective regulation tactic to decrease parental distress and improve parenting quality [19,20,21]. Higher parental self-compassion has been found to be related to lower parental anxiety [19,22], lower parental depression [19] and lower parental distress [20,22].

Social support is “a network of family, friends, neighbors, and community members that is available in terms of the need to give psychological, physical, and financial help” by the National Cancer Institute’s Dictionary of Cancer Terms. Social support can alter the health consequences of stressors in at least two ways. According to Cohen and Wills (1985) [23], social support can play a significant role in stress buffering, which refers to the protective role of social support in the relationship between stress and health. They suggest that social support protects individuals from the potential negative effects of stressful events. For instance, it can provide a sense of belonging and security, thus reducing the perceived impact of stress [23]. Moreover, Uchino [24] found that social support could also affect health through main effect models, which posit that social support has a direct and positive effect on health, regardless of the level of stress. This can be through various mechanisms such as influencing healthier behaviors, enhancing self-esteem, and providing a sense of meaning in life [24]. In both of these ways, social support can serve as a crucial resource in modifying or mitigating the health consequences of stressors.

Therefore, another objective of this study is to investigate how self-compassion and social support influence parenting stress experienced by caregivers of DHH children. It was hypothesized that self-compassion and social support can play a significant role in reducing parenting stress.

## 2. Materials and Methods

The study consisted of two stages: the first stage involved test translation and a pilot study, while the second stage focused on the psychometric characterization of the test examination and the identification of factors contributing to parenting stress.

### 2.1. Stage One: Test Translation and Pilot Study

Permission to adapt and validate the FSS for cross-cultural use in the Chinese population was granted by the questionnaire’s original developer [6]. The translation procedure adhered to the guidelines set out by Hall et al. [25]. Two bilingual translators, both native Mandarin speakers, were enlisted and given specific instructions by the first author regarding translation requirements and terminology. They independently produced written records of forward translations, combined into one forward translation document. No significant discrepancies were found between the two versions. A third bilingual translator was then brought in to produce a back-translation document, without having seen the original questionnaire. An expert committee, which consisted of a psychologist and two audiologists familiar with the target culture, was asked to review both the forward and back-translations of the questionnaire and finalize the translation. Only four wordings differed between the forward and back translations. However, the expert committee deemed these differences to be insignificant.

#### Pilot Study

A preliminary pilot study was carried out to identify any potentially confusing elements, words, or meanings in the questionnaire. This also enabled the gathering of suggestions from respondents on potential improvements. Moreover, an examination was conducted on how participants engaged with the instrument, with a particular focus on their responses to the format and length of the questionnaire. During the pilot phase, online questionnaires were distributed to 20 participants. No significant issues regarding confusing elements, words, or meanings emerged during the pilot study. The estimated time to complete the instrument was between 5 and 10 min. The instrument also demonstrated strong internal consistency, with a Cronbach’s alpha of 0.90.

### 2.2. Stage Two: Psychometric Characteristics of the CFSS and Factors Contributing to Parenting Stress

#### 2.2.1. Participants

To achieve 90% power and a Type I error rate of 0.05 while identifying variables with small effect sizes, a sample size of 245 participants is required for the multiple linear regression analysis with 11 independent variables. The study included 257 caregivers of children aged 0 to 12 years, with an average child age of 6.16 years (SD = 3.10), thereby meeting the sample size requirement. These children were recruited from rehabilitation centers and audiological clinics.

Among the children, 75.7% were fitted with hearing aids, with 1.9% receiving unilateral fittings and 73.7% bilateral fittings. The remaining 24.3% used cochlear implants, with 20.5% using one hearing aid along with one cochlear implant, 3.1% using two cochlear implants, and 0.8% having one cochlear implant without an additional hearing device. All participants used oral mode of communication. Additionally, 16 participants reported having other impairments: 8 had visual impairments, 6 had intellectual disabilities, 1 had ADHD, and 1 had Tourette syndrome.

The mean age at which devices (hearing aids for the HA group and cochlear implants for the CI group) were fitted was 3.10 years (SD = 2.29) for the left ear and 3.26 years (SD = 2.52) for the right ear. The mean duration of device use was 3.10 years (SD = 2.50) for the left ear and 2.95 years (SD = 2.48) for the right ear. The primary caregivers of most children were mothers (80.5%), followed by grandparents (14.3%) and fathers (4.5%). Approximately half of the primary caregivers (48.6%) held a university degree or higher. The primary caregivers were invited to complete an online survey, which took an estimated 30 min to complete and included various measures.

#### 2.2.2. Measures

Hearing Ability. The Categories of Auditory Performance-II [26] was employed to assess hearing functionality in daily life. This measure yields a standardized score across 10 ordinal categories, ranging from “no awareness of environmental sounds” (scored 0) to “using the telephone with an unknown caller in unpredictable situations” (scored 9). Research has documented high inter-rater reliability, with an overall agreement rate of 76% across evaluators [27].

Speech Intelligibility. The Speech Intelligibility Rating Scale [28] is a subjective assessment tool to evaluate how clearly speech is understood. Caregivers rate a child’s speech from the lowest level (Level 1) of being unintelligible to the highest level (level 5) of being easily understood by all listeners. Focused on overall intelligibility rather than parsing specific articulation errors, this scale is ideal for rapid evaluations in clinical or research contexts. Good inter-rater reliability has been reported, with interclass correlation coefficients ranging from 0.80 to 0.97 [29].

Oral Language Use. The Meaningful Use of Speech Scale, initially developed by Robbins in 1990, is a specialized tool designed to evaluate oral language usage among DHH children [30]. Consisting of 10 questions, the Meaningful Use of Speech Scale assesses a variety of vocal and speech-related behaviors. The questions are divided into several categories: vocalizing behavior (items 1–3), speech usage in family environments (items 4 and 5), application of oral skills in social contexts (items 6 and 7), the proportion of speech understood by others (items 8 and 9), and the child’s use of oral clarification skills (item 10). Caregivers are asked to rate their child’s daily speech behavior based on the observed frequency of these behaviors. Each item is rated on a five-point scale, with 1 indicating the behavior is never exhibited and 5 denoting consistent usage. The test—retest reliability of this scale is 0.93, and its Cronbach’s α is 0.89 [31].

Social Support. It was measured using the Multi-Dimensional Scale of Perceived Social Support [32]. It consists of 12 items and evaluates three sources of support: significant other, family and friends. Each item is answered on a 7-point Likert-type scale ranging from 1 = strongly disagree to 7 = strongly agree. It has been standardized in Chinese with good reliability and validity. The Cronbach alpha coefficients for the three subscales were: Family: 0.86; Friends: 0.89; Significant Other: 0.85. Additionally, test–retest reliability of 0.85 has also been obtained [33].

Self-Compassion. The Self-Compassion Scale [18] was used to evaluate self-compassion. It consists of 26 items, each rated on a 5-point Likert type scale ranging from 1 = almost never to 5 = almost always. It has been standardized in Chinese with good psychometric properties, including Cronbach’s α coefficients (0.84) and test–retest reliability (0.89) [34].

Self-compassion is a multifaceted construct that incorporates both positive and negative aspects. Positive aspects of self-compassion include self-kindness, common humanity, and mindfulness, while negative aspects include self-judgment, isolation, and over-identification. Two separate scores were utilized to inform the design of future interventions: one for the positive aspects of the Self-Compassion Scale (SCS-PA) and one for the negative aspects of the Self-Compassion Scale (SCS-NA).

General Parenting Stress. The Chinese Parental Stress Scale [35] was used to measure general parenting stress. It was adapted from the Parental Stress Scale developed by Berry & Jone [36]. The Scale focuses on stress as a reaction to stressor and keeps it conceptually different from the sources of stress. One item (there is little or nothing I wouldn’t do for my child(ren) if it were necessary) was excluded from the Chinese Parenting Stress Scale due to poor correlation [35]. It resulted in a total of 17 items, answered on a six-point Likert response scale ranging from 1 (strongly disagree) to 6 (strongly agree). Cronbach’s α coefficients of the Chinese Parenting Stress Scale were 0.892.

DHH-Related Parenting Stress. The CFSS, translated at the first stage, assessed the context-specific stress related to parenting a DHH child. The FSS comprises 16 items that evaluate general family stressors, such as safety and marital relationships, and stressors specifically associated with children who are DHH, such as communication issues and managing hearing aids or cochlear implants. Each item is rated on a 5-point Likert-type scale ranging from ‘not at all stressful’ to ‘extremely stressful’. The scale demonstrated strong reliability, with an internal consistency coefficient of 0.9.

### 2.3. Data Analysis

Psychometric Characteristics. The convergent validity of the CFSS was established by identifying correlations with the Chinese Parental Stress Scale using the Pearson product-moment correlation coefficient. Discriminate validity was evaluated by identifying a correlation between the scores on the CFSS and the Self-Compassion Scale. Previous studies suggested that individuals with higher levels of self-compassion tended to report less parenting stress [19,20,21]. Therefore, it was hypothesized a negative correlation between the Self-Compassion Scale and CFSS. The internal consistency reliability of the translated instrument was evaluated by calculating Cronbach’s alpha. Additionally, the intraclass correlation coefficient (ICC) was utilized to determine the test–retest reliability coefficient.

Factors Contributing to Parenting Stress. A hierarchical regression analysis (IBM SPSS version 19) was employed to identify factors that contribute to parenting stress measured using CFSS. In the first model, only device-related factors, such as the type of device (cochlear implant = 0, hearing aid = 1), age at hearing aid fitting/cochlear implant, and duration of device use, were included. The child’s hearing and communication factors, including hearing scale, speech intelligibility, and oral language use, were added to the second model. The third model incorporated caregiver-related factors, such as caregivers’ age, education level, self-compassion-positive aspects, and self-compassion-negative aspects. Lastly, social support was added in the fourth model.

The SPSS PROCESS macro was then employed to investigate how self-compassion and social support lessen parenting stress. In the moderation model, the primary challenges of DHH children—hearing and communication factors—were considered as the predictor, and parenting stress was considered as the dependent variable. Self—compassion and social support served as moderators. This interaction is deemed as moderation if it significantly predicts the dependent variable, in this case, parenting stress.

## 3. Results

The skewness values of the variables used in the subsequent analysis ranged from −0.98 to +0.99. The kurtosis values of these variables were between −1.38 and +1.59. According to George et al. [37] and Hair et al. [38], values for skewness and kurtosis falling between −2 and +2 are regarded as acceptable for demonstrating a normal univariate distribution. Since the skewness and kurtosis of the variables fell within these ranges, the data was thus considered to be normally distributed. Table 1 presents the mean, maximum possible score, standard deviation (SD), and range for these measures.

### 3.1. Psychometrics Characteristics

**Figure 1:**
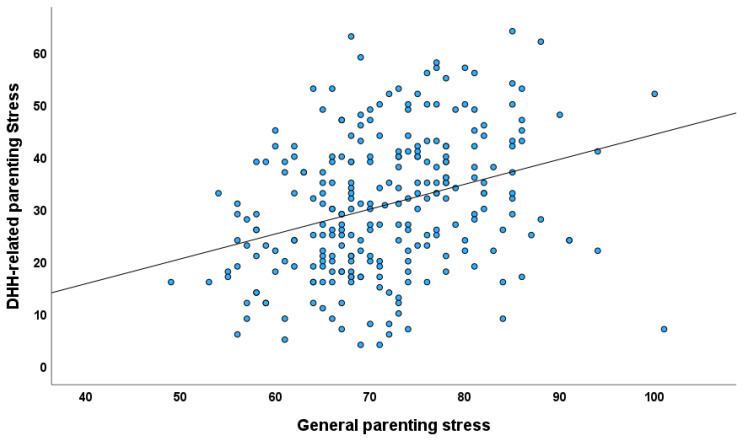
Scatterplot of general parenting stress measured vs. DHH-related parenting stress.

A positive correlation was found between the scores on the CFSS (mean = 30.71, SD = 13.20) and the Chinese Parental Stress Scale (mean = 71.56, SD = 8.94) (see Figure 1), with a correlation coefficient of 0.32 (*p* < 0.001). This suggests that patients experiencing higher levels of DHH-related parenting stress also showed elevated levels of general parenting stress. Furthermore, there was a negative correlation between the scores on the CFSS and the Self-Compassion Scale (mean = 82.11, SD = 11.59), with a correlation coefficient of −0.40 (*p* < 0.001). This indicates that patients with higher self-compassion reported lower levels of DHH-related parenting stress.

The CFSS also demonstrated strong internal consistency, with alpha coefficients at 0.93. High item-total correlations, ranging from 0.53 to 0.81, were observed for items in the CFSS, indicating a high degree of reliability among the responses. The test–retest reliability of the CFSS, as measured over a two-week interval, was also strong with an ICC of 0.93.

### 3.2. DHH-Related Parenting Stress

**Figure 2:**
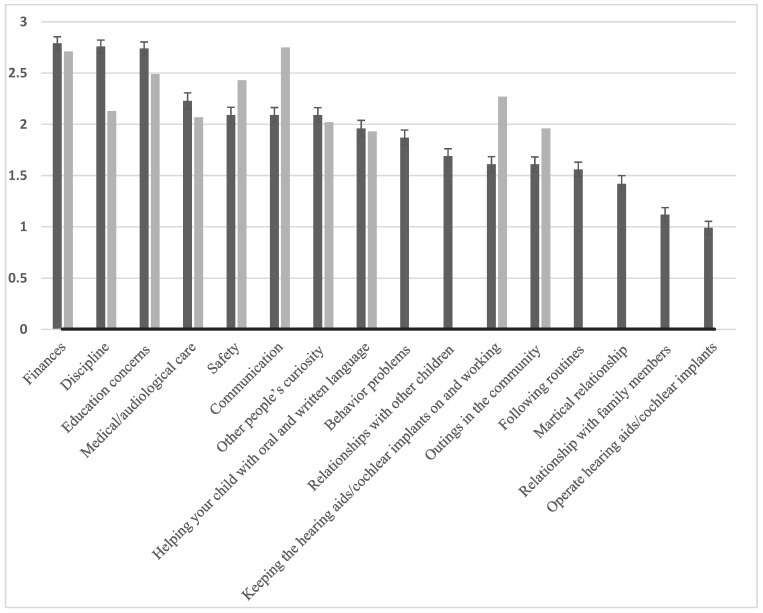
Illustrates a comparison between the top stressors for Chinese caregivers (represented by black bars) and English caregivers (represented by gray bars), as derived from Quittner et al. [12]. Note that only the top ten stressors for English caregivers were provided in Quittner et al.’s study.

The highest-ranked stressors for Chinese caregivers were (1) financial, (2) discipline, (3) education concerns, (4) medical/audiological care, (5) safety, and (6) communication (see Table 2). Compared to English speakers [12], Mandarin speakers demonstrated more concerns about discipline, behavior problems, relationships with other children and fewer concerns about communication, keeping the hearing aids/cochlear implants on and working and outings in the community (Figure 2).

### 3.3. Factors Affecting DHH-Related Parenting Stress

Hierarchical linear regression showed that device-related factors (i.e., Device type (cochlear implant = 0, hearing aid = 1), age at hearing aid fitting/cochlear implant, and duration of device use] accounted for significant variance in DHH-related parenting stress, R^2^ = 0.06. *F*(3, 245) = 4.84, *p* < 0.01. Both the age at which a hearing aid or cochlear implant was fitted, and the duration of device use showed a significant association with parenting stress, though they only explained 6% of the variance in parenting stress.

In the second model, the child’s hearing and language skills (i.e., hearing ability, speech intelligibility, and oral language use) were entered into the equation, accounting for significant variance over and above the previous set [ΔR^2^ = 0.16, Δ*F*(3, 242) = 15.93, *p* < 0.001]. Enhanced speech intelligibility and oral language use were linked to reduced levels of parenting stress, while the age at which a child received a hearing aid or cochlear implant and the duration of device use were no longer associated with parenting stress. This model explained a total of 21% of the variance in parenting stress.

In the third model, caregiver-related factors were entered into the equation to account for significant variance over and above the device and child’s hearing and communication [ΔR^2^ = 0.24, Δ*F*(5, 237) = 25.65, *p* < 0.001]. Self-compassion -negative aspects had a significant and negative relationship with parenting stress in this model, suggesting that those with low levels of self-compassion had more parenting stress related to DHH. Self-compassion -negative aspects accounted for an additional 24% of the variance in parenting stress.

Finally. Social support was added to model four and was significantly related to DHH-related parenting stress. In the final model, speech intelligibility, oral language use, self-compassion -negative aspects, and social support significantly contributed to 46% variances in parenting stress (see Table 3).

### 3.4. Moderated Analysis

A dual moderation analysis (PROCESS Model 2) was conducted to examine the effects of oral language use (X) on DHH-related parenting stress (Y), moderated by self-compassion-negative aspects (W) and social support (Z). The overall model was significant, R^2^ = 0.43, *F*(5, 251) = 38.63, *p* < 0.001. The interaction between oral language use and self-compassion-negative aspects (scores reversed, meaning higher scores reflect greater self-compassion with less self-judgment, isolation, and over-identification) was significant (b = −0.02, t = −2.10, *p* = 0.04, 95% CI [−0.037, −0.001]). This indicates that self-compassion -negative aspects moderated the effect of oral language use on DHH-related parenting stress. Specifically, parents with higher self-compassion experienced greater stress reduction when their child demonstrated better oral language use skills (b = −0.34, *p* < 0.001) compared to those with lower self-compassion (b = −0.34, *p* < 0.001). The interaction between oral language use and social support was not significant (b = 0.01, t = 1.08, *p* = 0.28), and the three-way interaction was also non-significant (*p* = 0.10). These findings suggest that self-compassion-negative aspects amplifies the stress-reducing effect of oral language use, while social support has a limited moderating role.

Another second dual moderation analysis (PROCESS Model 2) was conducted to examine the effects of speech intelligibility (X) on DHH-related parenting stress (Y), moderated by self-compassion—negative aspects (W) and social support (Z). The overall model was significant, R^2^ = 0.42, *F*(5, 251) = 36.08, *p* < 0.001, accounting for 41.82% of the variance in parenting stress. The main effect of negative self-compassion was significant (b = −0.97, t = −4.76, *p* < 0.001, 95% CI [−1.37, −0.57]), indicating that higher levels of self-compassion were associated with reduced parenting stress. However, the interaction between speech intelligibility and self-compassion-negative aspects was not significant (b = 0.05, t = 0.89, *p* = 0.38, 95% CI [−0.06, 0.15]), nor was the interaction between speech intelligibility and social support (b = −0.003, t = −0.07, *p* = 0.95, 95% CI [−0.08, 0.08]). The three-way interaction was also non-significant (*p* = 0.67). These results indicate that neither self-compassion-negative parts nor social support significantly moderates the relationship between speech intelligibility and DHH-related parenting stress.

## 4. Discussion

Parenting stress is crucial for the wellbeing of both children and caregivers [39]. Many studies have employed generic measures of parenting stress for caregivers of children with hearing loss. This approach may be inadequate as it overlooks the unique stressors and challenges faced by caregivers of DHH children. For example, we found that the top-ranking stressors for Chinese parents were (1) financial concerns, (2) discipline, (3) education concerns, (4) medical/audiological care, (5) safety, and (6) communication (refer to Table 1). Compared to English speakers, Mandarin speakers revealed more concerns about discipline, behavior problems, relationships with other children, and fewer concerns about communication, maintaining the operation of hearing aids/cochlear implants, and community outings.

Chinese, as a collectivist society, place a high value on discipline and social decorum, often emphasized from a young age. Consequently, parents might prioritize their children’s behavior and interpersonal relationships [40]. In contrast, Western cultures, characterized predominantly by individualism and expressiveness, may place more emphasis on communication and community interaction [40]. Thus, English-speaking parents might express more concern regarding their children’s communication skills and their capacity to function within society. Furthermore, due to the prevalent stigma surrounding deafness and a lack of deaf communication in China [41,42,43], most parents opt for oral mode of communication for their children, investing heavily in speech rehabilitation [14,15]. This could result in fewer communication concerns for these children in China. Additionally, the scarcity of professional audiology services and support forces parents to self-learn and manage the operation and maintenance of their child’s electronic devices [14,15,44]. Over time, these parents become adept at handling these devices and troubleshooting any issues, thereby reducing stress from this perspective in China. The findings align with Warner-Czyz et al. [17], which suggests that restricted access to audiological services, cultural perceptions of hearing loss, and divergent parental expectations could account for cross-national variations in parental assessments of quality of life.

Consequently, we developed the CFSS which demonstrated strong internal consistency and test–retest reliability. The analysis of the item-to-total relationships also showed strong correlations, affirming the conceptual foundations of the overall CFSS. The CFSS and the Chinese Parental Stress Scale showed a moderate relationship, implying that while they are related, the CFSS can independently reflect unique stressors experienced by caregivers of DHH children. Therefore, we propose that the CFSS is a reliable and valid tool for effectively measuring parenting stress among caregivers of DHH children.

Additionally, Wiseman et al. [13] compared parental stress levels in caregivers of school-age children and adolescents before and after cochlear implantation using the FSS. Their findings showed no significant overall difference in parenting stress pre- and post-implantation. However, specific domains of parenting stress—including keeping the HA/CIs on and working, safety, and communication—showed significant post-implantation reductions, which align with the lower stress scores in these same areas observed in our study compared to Quittner et al. [12].

We have further examined the impacts of various factors related to the device, such as the device type, the age at which a child receives a hearing aid or cochlear implant, and the duration of device use. We also considered the child’s hearing and communication skills (hearing skills, speech intelligibility, and oral language use), and caregivers’ factors, including caregivers’ age, education level, self-compassion, and social support, in understanding the variations in caregivers’ parenting stress related to DHH children. Our results showed that better speech intelligibility, better oral language use, lower self-compassion -negative aspects, and better social support were significantly associated with lower level of parenting stress of DHH children.

However, we did not find significant differences in parental stress between those using hearing aids and those using cochlear implants. This contrasts with the findings of Portelli et al. [45], who reported that parents of children with cochlear implants experienced higher stress levels than those whose children used hearing aids. Two factors might explain this discrepancy: first, Portelli et al. [45] measured general parenting stress using the PSS, which is not specific to parents of children who are DHH, whereas we used the CFSS. Additionally, as demonstrated in the hierarchical linear regression analysis, factors such as speech intelligibility, language abilities, self-compassion, and social support had a greater impact on parenting stress than the type of device used. These factors played a more crucial role in explaining the variance in parenting stress levels. Therefore, the differences in parenting stress between hearing aids and cochlear implants may be attributed more to variations in children’s speech and language skills, parents’ self-compassion, and the level of social support, rather than the device itself.

Hearing children with delayed language abilities often face additional hurdles due to their incapability to meet the escalating developmental demands of early childhood [12]. This problem is intensified when the child is also DHH. The stress experienced by caregivers of DHH children is related to the discrepancy between these developmental expectations and the child’s ability to meet them [8]. Moreover, adjusting their behavior and expectations to accommodate their child’s unique communication needs can pose a challenge to parents. Pipp-Segel et al. [10] revealed that parents who reported their children having lower language skills also reported experiencing higher levels of parenting stress. This may account for the significant relationship between language ability (i.e., speech intelligibility and oral language use) and parenting stress.

According to the Family Adjustment and Adaptation Response Model, families maintain a balance between demands (stressors, strains, and daily hassles) and capabilities (resources and coping strategies) [46]. When a family encounters a stressor such as a child’s deafness or hard-of-hearing, their equilibrium is disrupted, necessitating adjustments and adaptations to restore balance. We, therefore, examined self-compassion -negative aspects and social support as resilience factors in parenting stress and found that both significantly correlate with parenting stress.

Existing research suggests that parents with higher levels of self-compassion tend to experience less stress related to parenting [47]. This is because self-compassion offers a healthy way to manage negative emotions, making it easier to cope with the challenges associated with raising a DHH child [48]. Conversely, parents with lower levels of self-compassion may be more likely to experience high levels of stress, as they may be more critical of their parenting abilities and less able to cope with the difficulties they encounter [47].

It was also found that self-compassion-negative aspects (i.e., self-judgment, isolation, and over-identification) were significantly related to stress experienced by parents related to DHH, as opposed to self-compassion-positive aspects (i.e., self-kindness, common humanity, and mindfulness). These findings align with previous research suggesting that negative indicators are more strongly associated with mental health problems than positive indicators [49]. This is because negative aspects of self-compassion may reveal increased vulnerability or highlight harmful mechanisms contributing to mental health rather than the protective influence of self-compassion [49]. This valuable insight is crucial for designing interventions; interventions focusing on self-compassion-negative parts could potentially be more effective in alleviating stress in parents of DHH children.

Social support, which can come from various sources such as family, friends, and professional services, provides emotional, instrumental, and informational assistance, helping to alleviate stress among parents [8]. This support acts as a buffer against stressors, aiding parents in managing the complexities and challenges of parenthood (Cohen & Wills, 1985) [23]. Conversely, a lack of social support can escalate feelings of isolation and stress, leading to increased parenting stress [8]. Therefore, social support plays a critical role in managing parenting stress, fostering positive parent–child relationships, and enhancing overall family outcomes [50] (Belsky, 1984).

Given the significant roles of communication skills (i.e., speech intelligibility and oral language use), self-compassion-negative aspects (i.e., self-judgment, isolation, and over-identification), and social support in parenting stress, we further examined the role of self-compassion-negative aspects and social support in relationship between communication skills and parenting stress. Our results suggest that self-compassion amplifies the stress-reducing effect of improved oral language use. Specifically, parents with higher self-compassion (e.g., self-judgment, isolation, and over-identification) experience greater stress relief when their DHH child demonstrates better oral language skills compared to parents with lower self-compassion. These results underscore the importance of integrating self-compassion training into support programs for parents of DHH children to enhance the benefits of improved communication and effectively reduce parenting stress.

While we found a significant correlation between social support and parenting stress, social support does not seem to significantly moderate the relationship between communication skills (i.e., oral language use and speech intelligibility) and parenting stress. This suggests that while social support can provide emotional comfort and practical assistance, they may not directly mitigate the challenges and stressors caregivers face regarding their child’s oral language use or speech intelligibility. This leads us to infer that there may be alternative pathways through which social supports influence parenting stress, which warrants further investigation.

## 5. Limitations

Although this study utilized a relatively large sample size and adopted a multi-faceted approach to exploring parenting stress—integrating device-related factors, child communication skills, caregiver self-compassion, and social support—it had several limitations. First, the top stressors were compared between Mandarin and English speakers using the data from Quittner et al. [12] due to their relatively large sample size. However, we did not control for differences among various generations of cochlear implant users, such as age at implantation and duration of device use, which could have affected the results between English and Mandarin speakers. Second, as with many studies on parenting stress, all child-related information in this study was acquired through parent-reported data, providing a solely parental perspective. Incorporating objective data—such as speech perception and language assessments—could enhance the validity of the findings. Additionally, although the current study focused on certain factors, it did not include or analyze all important child-related factors, such as temperament and behavioral problems, which can significantly affect parental stress [13]. Including these factors in future research could provide a more comprehensive understanding of parental stress dynamics.

## 6. Conclusions

This study illuminates the unique stressors experienced by caregivers of DHH children and the contributing factors of such stress. Our research findings underscore the importance of considering cultural factors, a child’s communication skills, and caregivers’ self-compassion and social support in understanding and managing parenting stress. The development and validation of the CFSS tool further provide a more precise and culturally sensitive measure to assess parenting stress for caregivers of DHH children. Given the significant roles of self-compassion and social support, it is crucial to incorporate strategies that enhance these resilience factors in intervention programs. This would foster caregivers’ ability to cope with the stresses and challenges of raising DHH children. Future research should further explore the pathways through which social support influences parenting stress. Ultimately, a comprehensive understanding of these factors will facilitate the development of more effective strategies and resources to support caregivers and promote better outcomes for DHH children.

## Figures and Tables

**Table 1:** Mean, Maximum possible score, Standard Deviation (SD), and Range for measures.

Skills Tested	Measure	Mean/Maximum Possible Score	SD	Range
Hearing Ability	The Children’s Home Inventory for Listening Difficulties	4.74/9	2.81	0–9
Speech Intelligibility	The Intelligibility in Context Scale	3.81/5	1.30	1–5
Oral Language Use	The Meaningful Use of Speech Scale	38.25/50	8.54	12–50
Social Support	the Multi-Dimensional Scale of Perceived Social Support	57.97/84	12.59	17–84
Self-Compassion	The Self-Compassion Scale	82.11/130	11.59	37–120
General Parenting Stress	The Chinese Parental Stress Scale	71.56/102	8.94	49–101
DHH-Related Parenting Stress	The Chinese Family Stress Scale	30.71/80	13.20	4–64

**Table 2:** Represents the Mean and Standard Deviation (SD) for each item of the Chinese Family Stress Scale. The items are arranged in descending order, based on the mean score.

Items	Mean	SD	Range
Finances	2.78	1.04	1–5
Discipline	2.76	1.00	1–5
Educational concerns	2.74	1.03	1–5
Medical/audiological care	2.23	1.25	1–5
Safety (crossing the street)	2.09	1.24	1–5
Communication (Understanding you, speaking)	2.09	1.20	1–5
Other people’s curiosity or lack of understanding about child’s deafness	2.09	1.18	1–5
Helping your child with oral and written language	1.96	1.28	1–5
Behavior problems	1.87	1.21	1–5
Relationships with other children	1.69	1.17	1–5
Keeping the hearing aids/cochlear implants on and working	1.61	1.22	1–5
Outings in the community (keeping track of child; managing child’s behavior)	1.61	1.17	1–5
Following routines (mealtimes, bedtime)	1.56	1.16	1–5
Marital relationship	1.42	1.29	1–5
Relationships with parents or extended family	1.12	1.09	1–5
Understanding how to operate hearing aids/cochlear implants (stopping feedback).	0.99	1.04	1–5

**Table 3:** Hierarchical linear regression results (Standardized coefficients Beta).

	Model 1 (R^2^ = 0.06)		Model 2 (R^2^ = 0.21) ΔR^2^ = 0.16 ***	Model 3 (R^2^ = 0.45) ΔR^2^ = 0.24 ***	Model 4 (R^2^ = 0.46) ΔR^2^ = 0.01 *
Device-related factors	Device type (hearing aid = 1, cochlear implants = 0)	0.08	0.09	0.04	0.04
	Age at hearing aid fitting/cochlear implant	−0.21 ***	−0.12	0.001	0.001
	Duration of device use	−0.13 *	0.01	−0.003	−0.001
Child’s hearing and communication	Hearing ability		−0.06	−0.01	−0.02
	Speech Intelligibility		−0.16 *	−0.17 **	−0.15 *
	Oral language use		−0.29 ***	−0.21 ***	−0.21 ***
Caregiver-related factors	Age			−0.02	−0.01
	Education level			0.05	0.05
	Self-compassion—positive aspects			0.02	0.05
	Self-compassion—negative aspects			−0.52 ***	−0.49 ***
Social factors	Social support				−0.12 *

Note: * *p* < 0.05, ** *p* < 0.01, *** *p* < 0.001.

## Data Availability

The data that supports the findings of this study are available on request from the corresponding author. The data are not publicly available due to privacy or ethical restrictions.

## Abbreviations

The following abbreviations are used in this manuscript:

CFSS	Chinese Family Stress Scale
FSS	Family Stress Scale
DHH	deaf and hard-of-hearing
ICC	Intraclass Correlation Coefficient
